# Long-term outcome and fertility results of intraplacental choriocarcinoma: a retrospective study of 14 patients and literature review

**DOI:** 10.1186/s13023-024-03199-6

**Published:** 2024-05-22

**Authors:** Yang Liu, Xiaochen Song, Hui Zhang, Fengzhi Feng, Jun Zhao, Junjun Yang, Tong Ren, Xirun Wan, Fang Jiang, Yuan Li, Yang Xiang

**Affiliations:** 1 Department of Obstetrics and Gynecology, Peking Union Medical College Hospital, Chinese Academy of Medical Sciences & Peking Union Medical College, National Clinical Research Center for Obstetric & Gynecologic Diseases, No. 1 Shuaifu Road, Dongcheng District, Beijing, 100730 China; 2 Department of Pathology, Peking Union Medical College Hospital, Chinese Academy of Medical Sciences & Peking Union Medical College, Beijing, China

**Keywords:** Intraplacental choriocarcinoma, Fertility results, Prognosis, Gestational trophoblastic neoplasia

## Abstract

**Backgrounds:**

Intraplacental choriocarcinoma (IC) is an extremely rare subtype of gestational choriocarcinoma. The long-term follow-up and reproductive outcomes of IC patients remain unclear. Here, we report a series of 14 cases and conduct a literature review to assess the fertility and recurrence results of this rare disease.

**Results:**

Fourteen patients with pathologically confirmed IC treated in Peking Union Medical College Hospital between January 2002 and July 2022 were included in this study. Half of them had metastatic IC and were treated by chemotherapy with or without surgery. Only 1 patient had chemoresistant disease, but she achieved complete remission after immunotherapy. The median follow-up time was 45.5 months (range 4-192), and no recurrence occurred. One metastatic IC patient who achieved remission after chemotherapy had a full-term delivery. Among the 5 patients with fertility demands, 3 abandoned their pursuit of pregnancy because of “fear and worry about choriocarcinoma recurrence”. We reviewed a total of 89 cases of IC in English and Chinese literature from 1963 to 2022, and only 5 cases with subsequent pregnancy were reported, all of them were nonmetastatic IC cases.

**Conclusions:**

IC is sensitive to chemotherapy and has good long-term remission and a low recurrence rate. Patients with metastatic or nonmetastatic IC can have good pregnancy results after treatment. Doctors should pay more attention to the psychology of these patients.

**Clinical trial registration:**

N/A.

## Background

Intraplacental choriocarcinoma (IC), first reported by Driscoll [1] in 1963, is a rare subtype of gestational choriocarcinoma in which choriocarcinoma is found within the placenta. The clinical manifestations are atypical and can be asymptomatic lesions confined to the placenta [2] or metastatic choriocarcinoma with both maternal and infantile involvement [3]. It may cause fetal complications such as fetomaternal hemorrhage, stillbirth and intrauterine growth restriction in the perinatal period [4]. The reported incidence of gestational choriocarcinoma is 1 per 50,000 normal pregnancies [5]. And pathologically diagnosed IC has been reported to account for only 2.3% of gestational choriocarcinoma cases [6], making it extremely rare. Due to the rarity of IC, sometimes it’s difficult to histologically differentiate it from another equally rare disease, chorangiocarcinoma [7].

Literature review indicates that there are fewer than 100 reported IC cases until now, and the long-term follow-up and reproductive outcomes of it remain unclear. Peking Union Medical College Hospital (PUMCH) is a center for the diagnosis and treatment of gestational trophoblastic neoplasia in China. This retrospective study systematically analyzed the medical records of all IC patients treated in our center over the past 20 years (2002–2022), aiming to explore the long-term outcome and fertility results of this rare disease.

## Methods

The medical records and long-term follow-up data of all patients with a pathologically confirmed diagnosis of IC at PUMCH were reviewed. Demographic data and information on the presenting symptoms, gestational week and fetal outcomes were obtained from the clinical records. In this study, IC was staged according to the revised International Federation of Gynecology and Obstetrics (FIGO) criteria for GTN and assigned FIGO scores [8]. Written informed consent was obtained from each patient, and the study was approved by the Institutional Review Board of PUMCH (K3862).

The statistical analyses were performed with SPSS version 22.0 (SPSS Inc., Chicago, IL, USA). Data are presented as the means and standard deviations (SD) or medians (ranges) for continuous variables and as frequencies (corresponding percentages) for categorical variables.

## Results

### Demographic and clinical characteristics of the 14 patients

A total of 2,150 women were diagnosed with gestational choriocarcinoma at PUMCH between January 2002 and July 2022. Fourteen were pathologically diagnosed with IC, including 2 previously reported cases [3, 9]. Therefore, IC accounts for 0.7% of gestational choriocarcinoma diagnoses made at our center.

The demographic and clinical characteristics of all 14 patients are presented below (Table 1). Among the 14 patients, 10 were of Han nationality, 2 were of Hui nationality, and 2 were of Manchu nationality. The median age at diagnosis was 33 years (range 26–44), with a median of 3 previous pregnancies (range 1–5). Three (21.4%) patients were diagnosed in the first trimester (pregnancy 8 to 11^+ 6^ weeks). The 3 patients had abnormally and significantly increased β-human chorionic gonadotropin (β-hCG) levels up to 600,000 mIU/ml, and IC was confirmed by postabortion pathology. One (7.1%) patient was diagnosed in the second trimester (pregnancy 12 to 27^+ 6^ weeks). The remaining 10 (71.4%) patients were diagnosed in the third trimester (pregnancy 28 to 41 weeks) or postpartum, in terms of delivery methods, 6 of them (60%) underwent cesarean section due to obstetric factors or fetal distress, the other 4 patients (including 2 with intrauterine fetal deaths) underwent vaginal delivery.

The symptoms included vaginal bleeding (6/14, 42.9%), fetomaternal hemorrhage (5/14, 35.7%), and hemoptysis (1/14, 7.1%). Among the 7 cases of nonmetastatic IC, the β-hCG level automatically dropped to normal postpartum levels within 12.9 ± 8.2 weeks. Among the 7 cases of metastatic IC, the common metastatic sites were the lung (7/7, 100%), uterus (3/7, 42.9%), intracranial region (1/7, 14.3%) and vagina (1/7, 14.3%). There was 1 case in which fetal choriocarcinoma was diagnosed first, followed by a diagnosis of maternal metastatic IC [3].

**Table 1:** Clinical characteristics of the 14 patients with IC

Variable^*^	*n* = 14
Age, years	33 (26–44)
Body mass index, kg/m^2^	23.9 ± 4.8
Previous pregnancy	
Gravidity	3 (1–5)
Parity	0 (0–2)
GTN history	1 (7.1%)
Gestational stage	
First trimester	3 (21.4%)
Second trimester	1 (7.1%)
Third trimester or postpartum	10 (71.4%)
Gestational complications	
Gestational hypertension	0 (0.0%)
GDM	1 (7.1%)
β-HCG at diagnosis	
>10 × 10^4^	8 (57.1%)
<10 × 10^4^	6 (42.9%)
Symptoms^**^	
Vaginal bleeding	6 (42.9%)
Macroscopic placental abnormalities	5 (35.7%)
FMH	5 (35.7%)
Asymptomatic	4 (28.6%)
Hemoptysis	1 (7.1%)
Stage of IC	
Stage I	7 (50.0%)
Stage II	0 (0.0%)
Stage III	6 (42.9%)
Stage IV	1 (7.1%)

^*^The data are presented as the means ± standard deviations, median (minimum, maximum), or count (%)

^**^Some patients have more than one symptom

*GTN* gestational trophoblastic neoplasia, *GDM* gestational diabetes mellitus, *FMH* fetomaternal hemorrhage

The treatment of the 7 patients with metastatic IC is shown in Table 2. All patients were treated with chemotherapy. After chemotherapy, 3 (42.9%) underwent surgical treatment. One patient (14.3%) had chemoresistant disease but achieved complete remission after programmed cell death ligand-1 (PD-L1) immunotherapy.

**Table 2:** The treatment of the 7 patients with metastatic IC

	Stage	Chemotherapy protocol	Surgical procedure	Long-term follow-up	Fertility results
Case 1	III	FAEV	None	NED at 192 months	None
Case 2	III	actinomycin D, FAV, EMA-CO	None	NED at 70 months	Pregnancy at 37 months after treatment
Case 3	IV	FAEV, EMA-CO, TE/TP, FAEV with MTX intrathecal, EMA-CO with MTX intrathecal, PD-L1	VATS Resection of pulmonary lesions	NED at 41 months	None
Case 4	III	5-FU, FAV, FAEV, EMA-CO	TAH due to uterine choriocarcinoma perforation	NED at 64 months	None
Case 5	III	FAEV, AE	LH due to slow decline in β-HCG after chemotherapy	NED at 50 months	None
Case 6	III	FAEV	None	NED at 40 months	None
Case 7	III	FAV, FEV	None	NED at 4 months	None

*NED* no evidence of disease, *TAH* total abdominal hysterectomy, *LH* laparoscopic total hysterectomy, *VATS* video-assisted thoracic surgery, *FAEV* floxuridine, actinomycin-D, etoposide and vincristine, *FAV* floxuridine, actinomycin-D and vincristine, *EMA-CO* etoposide, methotrexate, actinomycin D, cyclophosphamide and vincristine, *TP/TE* paclitaxel, cisplatin/paclitaxel and etoposide, *FEV* floxuridine, etoposide and vincristine, *AE* actinomycin-D and etoposide, *MTX* methotrexate, *5-FU* 5- fluorouracil, *PD-L1* programmed cell death ligand 1

### Perinatal outcome

Four cases were excluded because they were diagnosed in the first or second trimester, and the perinatal outcomes of the remaining 10 patients are summarized below (Table 3). There were 2 intrauterine fetal deaths. Among the other 8 patients, each with a live birth, 1 had a premature delivery (35 weeks of gestation), and 7 had full-term deliveries. One newborn baby with a jejunal mass was treated by surgical resection, and pathology confirmed that it was choriocarcinoma. Then, the baby was successfully treated with combined chemotherapy [3]. Five newborn babies were pale and had anemia, which was confirmed to be associated with FMH.

**Table 3:** Perinatal outcomes of the 10 patients

Variable	*n* = 10
Intrauterine fetal death	2 (20%)
Live birth	8 (80%)
FMH	5 (62.5%)
Premature delivery	1 (12.5%)
Infant with metastatic disease	1 (12.5%)

### Long-term outcomes and fertility results

The median follow-up time was 45.5 months (range 4-192), and there was no recurrence among the 14 patients. Of the 5 patients with fertility demands, 2 (40.0%) became pregnant again. One patient had fetal malformation at 11 weeks of gestation and underwent therapeutically induced labor. The other patient who had metastatic IC and received actinomycin D for 4 cycles, received FAV for 1 cycle, then changed to EMA/CO for 2 cycles and underwent 3 additional cycles for consolidation after achieving remission. She had a spontaneous intrauterine pregnancy that reached term at 37 months after treatment. No abnormality was found in the placental pathology examination, and the baby was healthy. The remaining 3 (60.0%) patients abandoned their pursuit of pregnancy because of “fear and worry about choriocarcinoma recurrence”.

### Literature review

We reviewed and summarized the reported cases of IC in English and Chinese Literature from 1963 to 2022, and a total of 89 cases have been reported [6, 9–14]. Of the 101 IC patients (plus the 14 cases in our center, excluding the duplicate cases), 51 (50.5%) had non-metastatic IC. Among them, 7 patients (13.7%) received prophylactic chemotherapy, 1 was lost to follow-up, and 1 (2.0%) patient relapsed with lung metastasis and was cured by multiagent chemotherapy. In particular, 1 patient had a second diagnosis of IC confirmed by histological examination of the placenta after a second pregnancy. Further investigations revealed no evidence of metastasis, and her β-hCG level spontaneously returned to normal after delivery.

Among the 50 (49.5%) patients with metastatic IC, 2 patients were lost to follow-up, 13 (26.0%) patients died during the follow-up period, and 35 (70.0%) patients achieved complete remission by chemotherapy with or without surgery/radiotherapy. We further analyzed the 13 patients who died and found that only 3 patients had received chemotherapy. The remaining 10 patients were from an era when chemotherapy was not developed, 5 patients received only surgery, 4 died prior to initiation of therapy, and there was 1 treatment-related death.

At present, only 5 cases with subsequent pregnancy have been reported, [6, 9, 15, 16] all of them were nonmetastatic IC cases, with good maternal and infant outcomes.

## Discussion

The fertility and recurrence results of IC at long-term follow-up were assessed in this study. We found that IC is sensitive to chemotherapy and has good long-term remission with a low recurrence rate, and patients with metastatic or nonmetastatic IC can have good pregnancy results after treatment.

The application of chemotherapy has greatly improved the prognosis of gestational choriocarcinoma patients. As a rare subtype, the main treatment for IC is also chemotherapy. Duleba et al. [17] recommended surveillance alone in nonmetastatic IC cases and chemotherapy for metastatic IC cases. A review published in 2013 mentioned that before chemotherapy was available, the survival rate at 5 years with hysterectomy alone was 41% in nonmetastatic IC and 19% in cases of metastasis [18]. However, almost all metastatic IC patients have achieved long-term remission since the application of effective multiagent chemotherapy [6]. In our study, there was one patient with chemotherapy resistance who received PD-L1 immunotherapy and achieved long-term remission. Based on our review of the literature and the results of our study, we recommend observation for nonmetastatic IC cases and combination chemotherapy for metastatic IC.

The reproductive outcomes of IC patients have rarely been discussed. All cases with subsequent pregnancy have been reported were nonmetastatic IC patients. For patients with metastatic IC, no subsequent pregnancy has been reported ever. In our study, it is noteworthy that full-term delivery occurred in a metastatic IC patient who achieved remission after chemotherapy. A review showed that after undergoing chemotherapy for GTN, 86.7% of women desired to conceive, the term live birth rate was 75.8%, and multiagent chemotherapy did not increase the risk of adverse obstetric events or the rate of fetal malformation in pregnancy [19]. Based on the aforementioned research, we believe that both metastatic and nonmetastatic IC patients were able to have good pregnancy outcomes.

It is worth noting that 60.0% of patients in our study with fertility demands abandoned the plan for later pregnancy because of “fear and worry about choriocarcinoma recurrence”, indicating that the psychological burden of IC patients should be an important consideration. Gestational choriocarcinoma is closely related to pregnancy events, including molar pregnancies, normal pregnancies, miscarriages, and ectopic pregnancies. Patients worry about the possibility of recurrence of gestational choriocarcinoma, and psychological morbidity rates exceed community levels even among patients who do not require chemotherapy [20]. Based on the current literature and the experience of our study, there is no clear evidence that subsequent pregnancy in patients with IC leads to IC recurrence. These can be used to support subsequent pregnancy considerations in patients with IC.

Our study has some unique strengths. This is the first report of patients with metastatic IC who had successful pregnancy after undergoing treatment with chemotherapy. Moreover, this is the first study to analyze the clinical features and prognosis of more than 10 IC cases. The primary limitation of the present study is that it is a retrospective study with a long enrollment period (20 years). However, because IC is an extremely rare disease, it is difficult to report more cases in a short time span.

## Conclusions

IC is a rare subtype of gestational choriocarcinoma that is sensitive to chemotherapy and has good long-term remission with a low recurrence rate. Patients with metastatic or nonmetastatic IC can have good pregnancy results after treatment. Doctors should pay more attention to the psychology of these patients.

## Data Availability

The datasets generated and analysed during the current study are not publicly available due the need to protect study participant privacy but are available from the corresponding author on reasonable request.

## Abbreviations

IC: Intraplacental choriocarcinoma
PUMCH: Peking Union Medical College Hospital
FIGO: International Federation of Gynecology and Obstetrics
β-hCG: β-human chorionic gonadotropin
GTN: gestational trophoblastic neoplasia
GDM: gestational diabetes mellitus
FMH: fetomaternal hemorrhage
NED: no evidence of disease
TAH: total abdominal hysterectomy
LH: laparoscopic total hysterectomy
VATS: video-assisted thoracic surgery
FAEV: floxuridine, actinomycin-D, etoposide and vincristine
FAV: floxuridine, actinomycin-D and vincristine
EMA-CO: etoposide, methotrexate, actinomycin D, cyclophosphamide and vincristine
TP/TE: paclitaxel, cisplatin/paclitaxel and etoposide
FEV: floxuridine, etoposide and vincristine
AE: actinomycin-D and etoposide
MTX: methotrexate
5-FU: 5-fluorouracil
PD-L1: programmed cell death ligand 1
